# Case report: Atypical and chronic masticatory muscle myositis in a 5-month old Cavalier King Charles Spaniel. Clinical and diagnostic findings, treatment and successful outcome

**DOI:** 10.3389/fvets.2022.955758

**Published:** 2022-09-14

**Authors:** Martin Di Tosto, Carolina Callegari, Kaspar Matiasek, Giuseppe Lacava, Giovanna Salvatore, Sara Muñoz Declara, Barbara Betti, Federica Tirrito

**Affiliations:** ^1^AniCura Istituto Veterinario Novara, Granozzo con Monticello, Novara, Italy; ^2^Section of Clinical & Comparative Neuropathology, Ludwig Maximilian University of Munich, Munich, Germany; ^3^Studio Veterinario Associato Vet2Vet di Ferri e Porporato, Orbassano, Torino, Italy

**Keywords:** CKCS, CT, dog, masticatory muscle myositis, MRI

## Abstract

Masticatory muscle myositis (MMM) is the second most common inflammatory myopathy diagnosed in dogs, but it is rarely described in puppies. The disease is characterized by the production of autoantibodies against 2M myofibers contained in masticatory muscle, although the cause of this production is still unclear. The aim of the present case report was to describe the clinical presentation, diagnostic findings, treatment, and follow-up of an atypical case of chronic masticatory muscle myositis in a very young dog. A 5-month old Cavalier king Charles Spaniel (CKCS) was presented to the AniCura Istituto Veterinario Novara with a two weeks, progressive history of lethargy and difficulty in food prehension. Neurological examination revealed bilateral masticatory muscle atrophy, mandibular ptosis with the jaw kept open, inability to close the mouth without manual assistance, jaw pain, and bilateral reduction of palpebral reflex and menace reaction; vision was maintained. A myopathy was suspected. Computed tomography (CT), magnetic resonance imaging (MRI), enzyme-linked immunosorbent assay test for 2M antibodies, and histopathological examination of masticatory muscle biopsy confirmed the diagnosis of MMM. Glucocorticoids treatment was started and clinical signs promptly improved. To the authors' knowledge, this is the first case describing mandibular ptosis in a dog affected by chronic MMM, successfully managed with medical treatment and the first report describing the CT and MRI findings in a young CKCS affected by MMM.

## Introduction

Masticatory and limb muscles have the same embryonic precursor and are composed by type 1 and type 2 myofibers (1). One feature of masticatory muscle is the presence of type 2M fibers and a type 1 fiber variant (1–6). However, the masticatory muscle formed by type 2M fibers and a type 1 fiber variant are the temporalis muscle, masseter muscle and pterygoid (lateral and medial) muscles; interestingly, digastricus muscle is the only masticatory muscle that does not have type 2M fibers and a type 1 fiber variant (4, 5, 7). Masticatory muscle myositis (MMM) is one of the most common inflammatory myopathy of the dog (3, 8). MMM have an immune-mediated origin in which autoantibodies against the type 2M fibers are produced. The etiology of this myopathy is still unknown, and one hypothesis is based on molecular mimicry with autoantibodies produced in response to an infectious agent that cross react with self-antigens of 2M myofibers (5). Dogs affected by MMM can be presented for lethargy, jaw pain, hyporexia or anorexia, fever, trismus (jaw muscles spasm with restriction in mouth opening), enophtalmos or exophthalmos, swelling or atrophy of the face (4, 5, 9). Swelling and pain of masticatory muscle are typical of the acute phase, whereas in the chronic phase muscle atrophy is prevalent and masticatory muscle are replaced by fibrous tissue (4). MMM can affect any dog, but a predilection for large breed dogs has been observed and in particular German shepherds, Golden retriever, Labrador retrievers, Doberman pinschers, and Cavalier King Charles Spaniels (CKCS) (4, 5).

The MMM has not an age predisposition (4); however, most dogs with MMM are young adults and the median age of onset is 3 years, but diagnosis of MMM has also been reported in CKCS puppies as young as 4-months (4, 5, 10). The serological test for 2M antibodies has a high sensitivity (80–90%) and specificity (100%) for diagnosis of masticatory muscle myositis and is the preferred diagnostic test (5). Diagnostic imaging with computed tomography (CT) and magnetic resonance imaging (MRI) may aid in diagnosis of MMM, revealing swelling in acute phase or atrophy in chronic phase of masticatory muscle, areas of masticatory muscle that present signal changes, and contrast enhancement (4, 11). Histological evaluation of masticatory muscle biopsy is another procedure that may aid in the diagnosis of MMM and, at the same time, is useful in providing information about prognosis (5). In the acute phase, muscle biopsy reveals mixed inflammatory infiltrates associated with myofiber necrosis and phagocytosis, while in the chronic phase muscle biopsies are characterized by fibrous tissue and low cellular infiltration (5, 12). Treatment of MMM is based on the use of immunosuppressive drugs and glucocorticoids are usually the first choice (5, 13). A positive outcome in MMM necessitates early diagnosis and appropriate treatment. In fact, patients treated in the chronic phase of the disease are affected by muscle fibrosis and may carry a worse prognosis, compared to acute phase, for improvement of clinical signs (5). The aims of this study were to report the clinical presentation of a 5-month old CKCS affected by an atypical and chronic form of MMM and to describe CT and MRI abnormalities and follow- up of this unusual case in order to better understand clinical and diagnostic alterations that may be found.

## Case description

A 5-month old, intact male CKCS was referred for a two weeks progressive history of lethargy, inability to close the jaw, and difficulty in food prehension. No information on littermates' health was available. Clinical examination revealed lethargy, and mandibular and retropharyngeal lymphadenopathy; no ocular abnormality (e. g. conjunctivitis, enophthalmos or exophthalmos) was present. Neurological examination revealed inability to close the mouth without assistance and concurrent inability to adequately open the jaw (trismus), jaw pain, bilateral reduction of palpebral reflex and menace reaction with incomplete closure of the eyelids; bilateral atrophy of temporal and masseter muscles was also present (Supplementary Video 1). The neurolocalization was neuromuscular; a myopathy was suspected but facial or trigeminal neuropathies could not be excluded because of the incomplete closure of the eyelids and the mandibular ptosis. Blood analysis, serology for infectious diseases (*Dirofilaria immitis, Ehrlichia canis, Leishmania infantum, Borrelia* spp., *Anaplasma phagocytophilum, Babesia canis, Hepatozoon canis, Toxoplasma gondii*, and *Neospora caninum*), fine needle aspiration of lymph nodes, 2M antibodies assay ELISA, CT and MRI of the head and biopsies of temporalis and masseter muscles were performed.

## Diagnostic assessment, therapeutic intervention, follow-up and outcome

**Figure 1:**
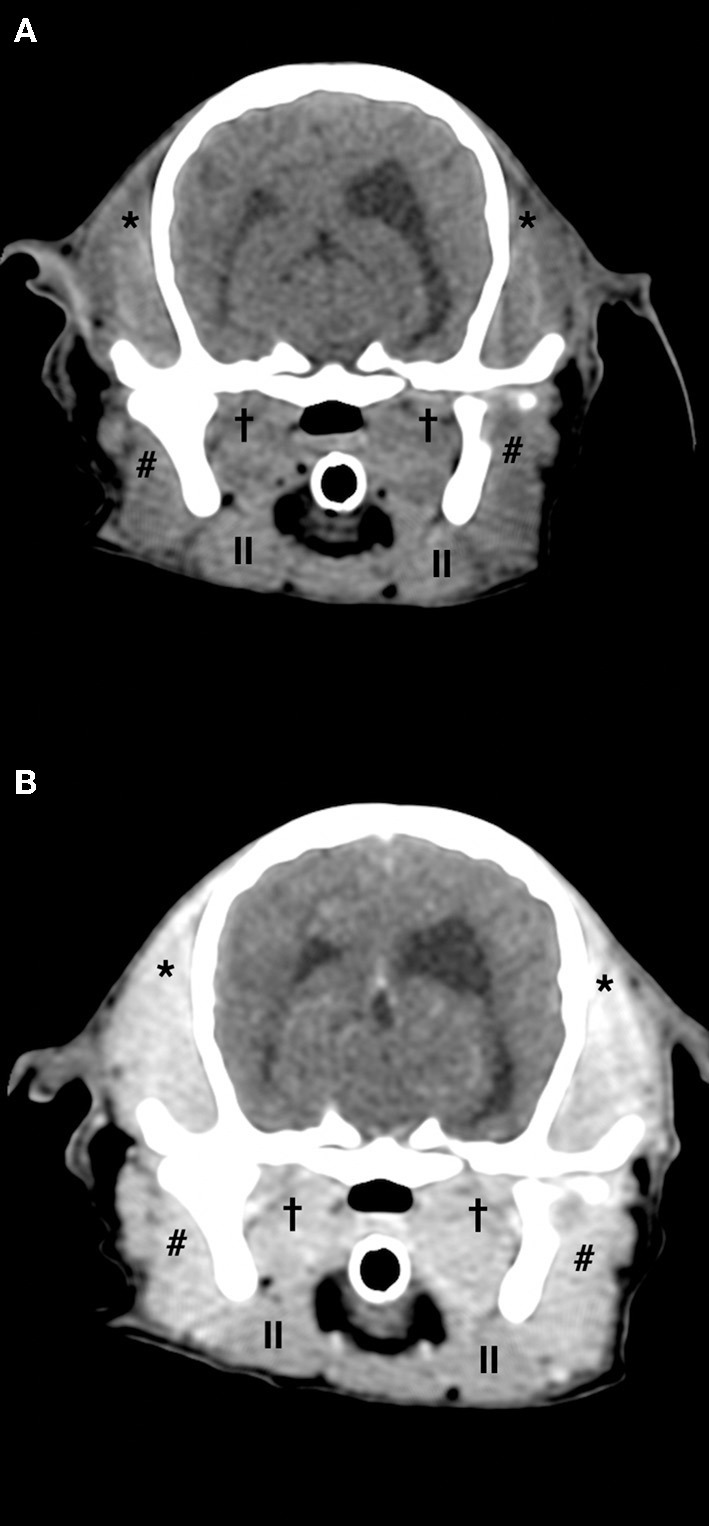
Computed tomography pre-contrast **(A)** and post-contrast **(B)** images of the head of a 5-month old Cavalier King Charles Spaniel affected by masticatory muscle myositis. Note the severe, diffuse, and bilateral atrophy of temporalis, masseter, and pterygoid muscles and contrast enhancement of the masticatory muscle in post-contrast image (* temporalis muscle, ^#^masseter muscle,^†^pterygoid muscle, ^||^digastricus muscle).

**Figure 2:**
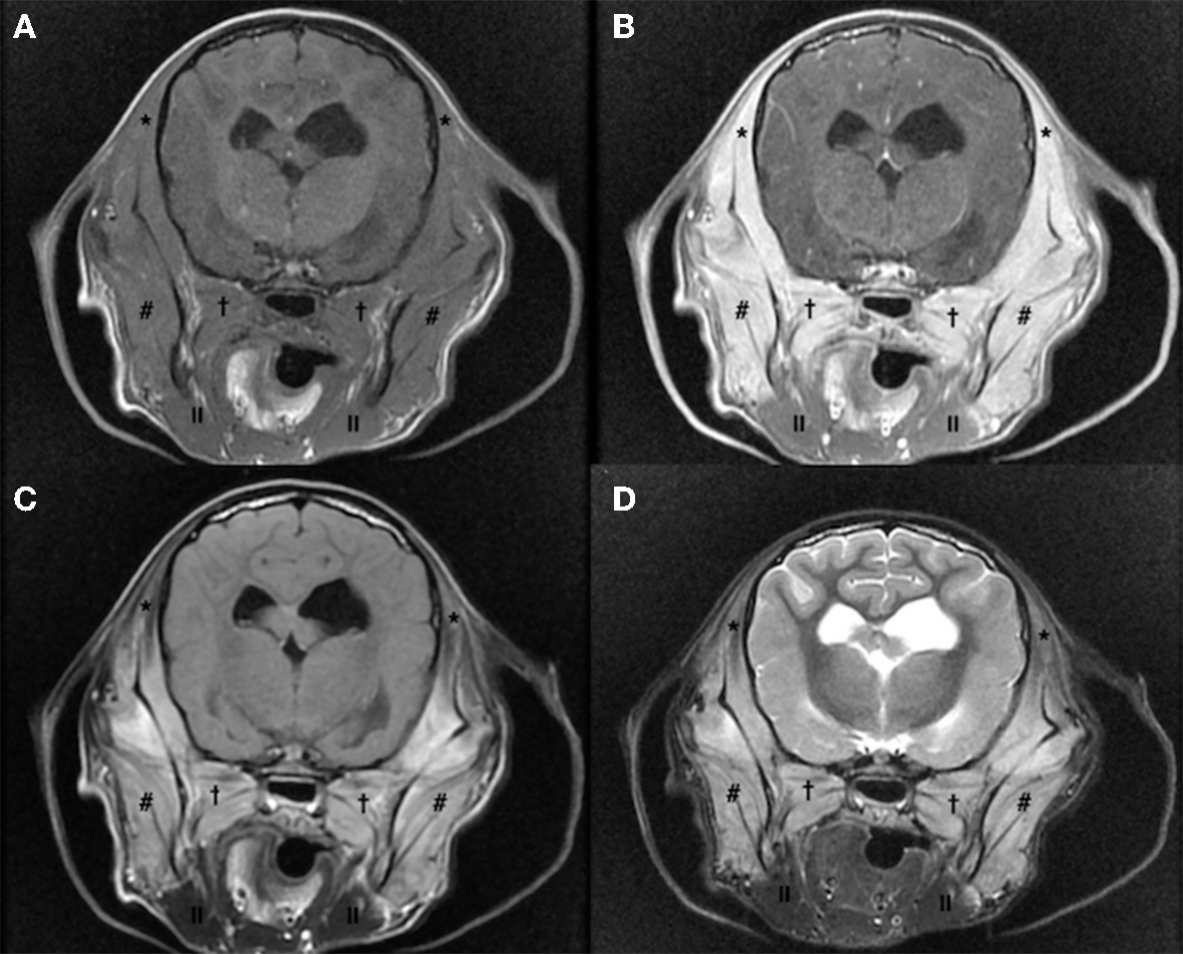
Magnetic resonance imaging study of the head of a 5-month old Cavalier King Charles Spaniel affected by masticatory muscle myositis; pre-contrast T1-W image **(A)**, post-contrast T1-W image **(B)**, fluid attenuated inversion recovery (FLAIR) **(C)** and short tau inversion recovery (STIR) **(D)** images. Note the severe, diffuse, and bilateral atrophy of temporalis, masseter, and pterygoid muscles that appear isointense on T1-W images pre-contrast **(A)** and showing a strong contrast enhancement **(B)**; these muscles appear hyperintense on FLAIR **(C)** and STIR **(D)** images, compared to the signal in normal muscles (* temporalis muscle, ^#^masseter muscle,^†^pterygoid muscle, ^||^digastricus muscle).

**Figure 3:**
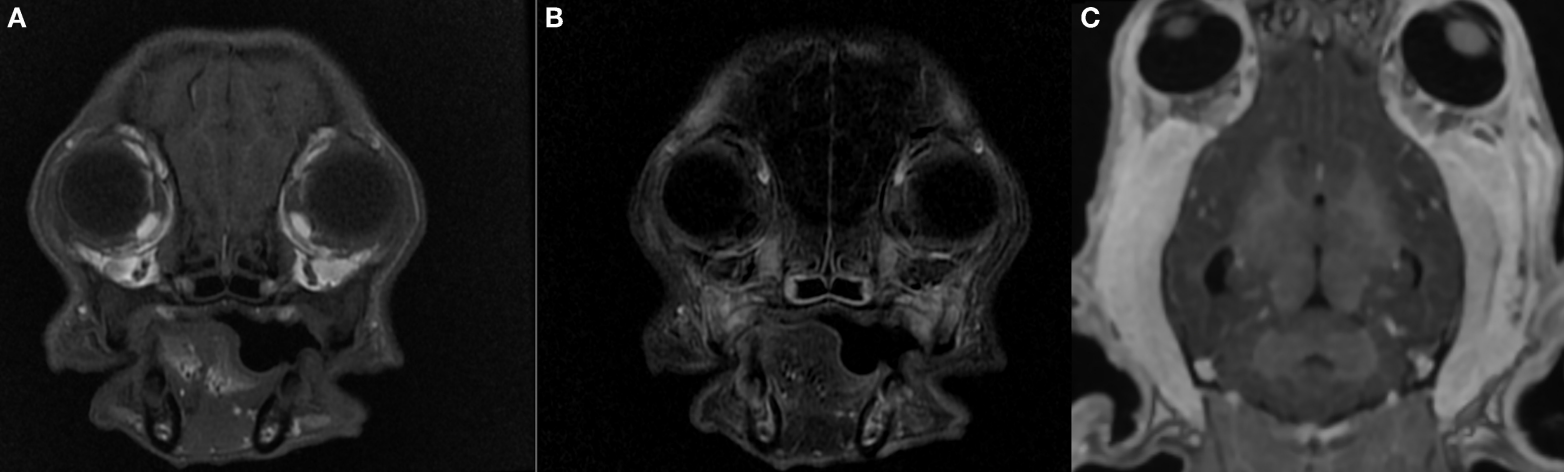
Magnetic resonance imaging study of the head of a 5-month old Cavalier King Charles Spaniel affected by masticatory muscle myositis; pre-contrast T1-W image **(A)**, post-contrast T1-W subtraction image **(B)**, pre contrast 3D T1-W image **(C)**. Note the absence of extraocular muscle swelling and contrast enhancement; no evidence of exophthalmos.

**Figure 4:**
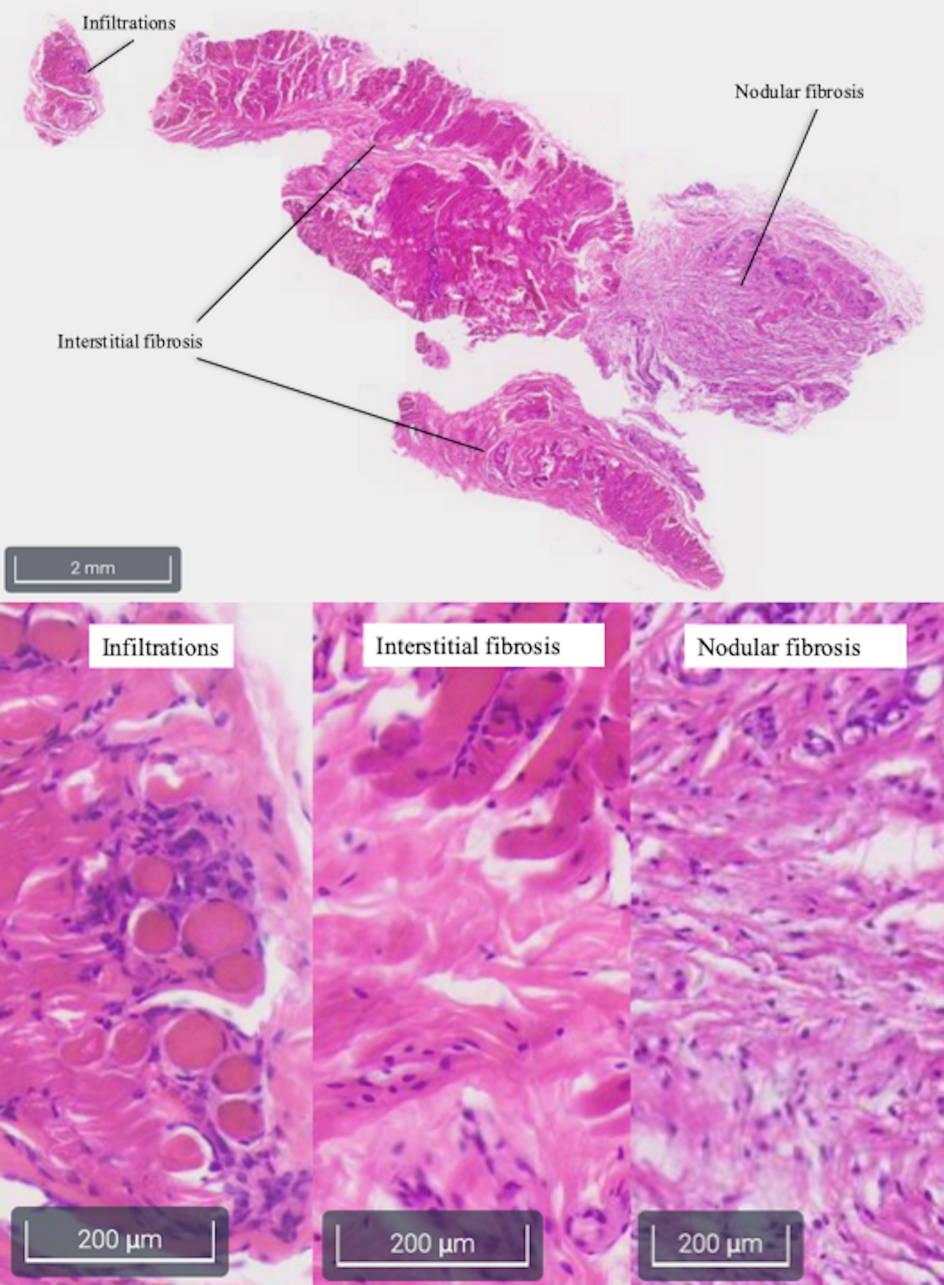
Photomicrograph of histological, hematoxylin and eosin stained, section of a temporalis muscle of a 5-month old Cavalier King Charles Spaniel affected by masticatory muscle myositis. Note the inflammatory infiltration and the interstitial and nodular fibrosis of the temporalis muscle.

Hematology and biochemistry evaluation revealed mild anemia (29.1% hematocrit, range 38.3–56.5%), moderate leucocytosis (33.4 K/μL, range 4.9–17.6 K/μL) with mature neutrophilia (28.1 K/μL segmented neutrophils, range 2.94–12.67 K/μL), mild hyperglobulinemia (4.9 g/dL, range 2.4–4.3 g/dL) and hypoalbuminemia (2.3 g/dL, range 2.8–4.3 g/dL), mild elevation of creatine kinase (2126 U/L, range 41–378 U/L), mild increase of aspartate aminotransferase (237 U/L, range 14–159 U/L), and mild elevation of C reactive protein (61.7 mg/L, range 0–10.7 mg/L). Trismus was confirmed because it was not possible to open the dog's mouth during general anesthesia. Lymph node cytology revealed reactive lymphadenopathy. Diagnostic imaging was performed and CT study (64 slices, Optima 660, General Electric, Milan, Italy) of the head highlighted symmetrical reduction in volume of masseter, temporalis, and pterygoid muscles with moderate contrast enhancement (Figure 1); mandibular and retropharyngeal lymphadenomegaly was also reported. Magnetic resonance imaging study (1.5 Tesla, SIGNA Creator, General Electric, Milan, Italy) of the head revealed symmetric and severe masticatory muscle atrophy. Temporalis, masseter, and pterygoid muscles showed diffuse, bilateral and heterogeneous hyperintensity on T2-weighted, fluid attenuated inversion recovery (FLAIR) and short tau inversion recovery (STIR) images; these muscles appeared isointense on T1-weighted sequences compared to the signal of normal muscles (Figure 2). A strong and diffuse contrast enhancement of the masticatory muscle (with the exception of the digastricus muscle) was present after contrast administration (Figure 2), no signal changes, contrast enhancement or swelling of extraocular muscle were identified (Figure 3); regional lymph nodes appeared enlarged. ELISA test for 2M antibodies was 1:1000 (reference range < 1:100). Serology for infectious diseases was negative with exception of mild positivity for *B. canis* (15.8 U, reference range < 14 U); serology for *B. canis* was repeated two weeks later and ruled out an acute infection. Temporalis and masseter muscle histopathology revealed multifocal, mixed cellular, chronic, moderate to marked myofascitis with myofibrosis and muscle atrophy (Figure 4). Based on clinical signs, imaging and histopathological findings, a diagnosis of an atypical and chronic form of juvenile MMM was made. Prednisolone was started with a dose of 1 mg/kg every 12 h. After two weeks of therapy the dog developed intense glucocorticoids associated side effects (i.e., gastrointestinal signs and severe neutrophilic leucocytosis); for this reason, glucocorticoids were rapidly tapered to 0.5 mg/kg every 24 h and this dose was continued for 3 weeks. At follow-up evaluations, clinical signs of MMM and blood analysis improved: dropped mandible and trismus were no longer present, food prehension was normal, masticatory muscle atrophy improved, and creatine kinase and c-reactive protein levels in serum were within reference range. The dose of prednisolone was further gradually tapered in three months. At three months follow-up, the dog was clinically stable and treatment with glucocorticoids was interrupted. Three months after treatment, the dog showed a mild relapse of clinical signs with reluctance to play with a ball due to difficulty in opening the mouth and mild inappetence; a course of prednisolone (0.5 mg/kg every 24 h) of one month duration was effective in complete resolution of clinical signs. Two months after the last administration of glucocorticoids, the dog did not present any signs of jaw pain or difficulty in food prehension, complete blood exams were unremarkable, and neurological examination was normal with the exception of moderate masticatory muscles atrophy (Supplementary Video 2). ELISA test for 2M antibodies was repeated and the result was negative (test result < 1:100, reference range < 1:100).

## Discussion

Masticatory muscle myositis in juvenile CKCS is a rare immune-mediated disorder for which a genetic predisposition has been hypothesized and the classical clinical signs of MMM are trismus, jaw pain and swelling or masticatory muscle atrophy (5). In our case, no information about littermates was known and in addition to the classical clinical signs, a reduction of palpebral reflex and menace reaction, and mandibular ptosis were also encountered. Deficit of palpebral reflex and menace reaction may be the result of orbicularis oculi muscle myopathy or facial neuropathy, anatomical structures that are generally spared in cases of MMM (10, 14). A previous case report of CKCS dogs affected by an atypical form of MMM reported the loss of palpebral reflex and menace reaction but MRI study was not performed and the etiology of those clinical signs remained unclear. Ocular signs have also been reported in 44% of dogs affected by MMM; it is because, in the acute phase, the swelling of pterygoid muscle may cause exophthalmos (5). In the present case the dog was affected by a chronic form of MMM and MRI study did not reveal swelling of pterygoid muscle or any abnormalities or signal changes of orbicularis oculi muscle (Figure 3). An electrodiagnostic investigation, not performed in our case, might have helped in clarifying the cause of these clinical signs.

Part of the uncommon clinical presentation of the dog was the concurrent trismus and inability to raise the mandible. Trismus refers to a masticatory muscle spasm causing a reduction in jaw movement (15) and is reported in 41% of dogs with MMM, representing one of the main clinical signs in this disease (9). In contrast, the inability to close the mouth without manual assistance has been previously reported only in one dog allegedly affected by MMM; specifically, the dog showed an acute form of masticatory muscle compartmental syndrome due to histiocytic and neutrophilic temporalis muscle myositis with myofiber degeneration and necrosis that caused masticatory muscle dysfunction and inability to close the mouth (16). The authors hypothesized that the compartmental syndrome was the consequence of the masticatory muscle myositis but the diagnosis of MMM was not confirmed since the ELISA test for 2M antibodies was negative (16). Therefore, to the authors' knowledge, this is the first report describing a dropped mandible in a case of confirmed MMM. A possible explanation for this peculiar clinical sign might be a bilateral neuropathy of the trigeminal nerve due to an extension of the disease from the masticatory muscle to the fifth nerve (10). However, this hypothesis was considered unlikely because of the absence of any other fitting clinical signs, besides from the dropped mandible, or of alterations of the trigeminal nerve and trigeminal nucleus reported at the neurological examination and CT and MRI studies, respectively. Another justification for the inability to close the jaw might be the reduced functionality of temporalis, masseter, and pterygoid muscles, whose main task is to close the mouth (17), as a consequence of their inflammation, atrophy, and fibrosis.

MMM has been scantly described in CKCS puppies (10) and previous reports describing CT and MRI findings in dogs affected by MMM have been mainly conducted in other breeds (11, 18, 19); in particular on CT scan, all masticatory muscle besides from digastricus muscle may appear reduced in volume as a consequence of muscle atrophy, and hypoattenuating in pre-contrast scans with inhomogeneous contrast enhancement; regional lymph nodes may appear enlarged with homogenous contrast enhancement (18). MRI however is considered the best imaging modality to identify MMM in dogs, allowing an early detection of the affected muscles (11, 20, 21). MRI findings include iso-hypointense signal of masticatory muscles on T1-weighted images and hyperintense signal of these structures on T2-weighted, FLAIR, and STIR images, compared to the signal of other muscles, contrast enhancement of masticatory muscle is often present (11, 16). CT and MRI findings of our case are similar to those described in the previous literature.

In the present case, glucocorticoids treatment allowed a significant clinical improvement, despite the signs of chronic inflammation. The dog was able to completely open its mouth and most of the masticatory muscle trophism recovered.

Chronic forms of MMM have generally a poor prognosis, based on muscle fibrosis and atrophy; however, restoration of masticatory muscle function has been reported (4, 22). In particular, few reports described young dogs affected by MMM with histopathological evidence of muscle fibrosis and atrophy that regained jaw mobility and masticatory muscle trophism after glucocorticoids treatment (10, 22). An hypothesis for the relevant improvement of clinical signs in young dogs affected by chronic MMM might be that the skeletal muscle resident stem cells have a regenerative capacity and are therefore able to respond to tissue injury, this regenerative capacity may decline with aging (23, 24). However, the relationship between histopathological findings and recovery of masticatory muscle function in younger compared to older dogs needs further investigation. A previous study on juvenile CKCSs affected by an atypical form of MMM also reported progressive clinical improvement and normalization of facial appearance with glucocorticoids; nevertheless, in that study dogs were presented for examination earlier in the course of the disease than the dog of this report (10).

This report described a case of CKCS puppy affected by an atypical form of MMM that showed trismus and concurrent mandibular ptosis and it is interesting to speculate that CKCS puppies may be affected by a breed-specific variant of MMM. To the authors' knowledge, there are no previous reports describing CT and MRI findings of juvenile chronic form of MMM in CKCS dogs and, based on the present case, it is possible that this form has a similar MRI appearance to the previously reported adult forms of MMM. Young CKCSs with MMM might have a good prognosis with rapid and marked improvement with glucocorticoid treatment, even if presented with signs of chronicity.

## Data availability statement

The original contributions presented in the study are included in the article/Supplementary material, further inquiries can be directed to the corresponding author/s.

## Ethics statement

Ethical review and approval was not required for the animal study because the authors presented the case of a dog that underwent standard clinical and diagnostic investigations (blood exams, CT and MRI studies of the head, muscle biopsy) approved by the owner and did not perform any experimental treatment. Written informed consent was obtained from the owners for the participation of the animal in this study.

## Author contributions

MD, CC, KM, GL, GS, SM, BB, and FT contributed to the conception and design of the study. CC and FT followed all of the clinical aspects of the case. MD and FT wrote the first draft of the manuscript. CC, KM, GL, GS, SM, and BB wrote sections of the manuscript. All authors contributed to the article and approved the submitted version.

## Conflict of interest

The authors declare that the research was conducted in the absence of any commercial or financial relationships that could be construed as a potential conflict of interest.

## Publisher's note

All claims expressed in this article are solely those of the authors and do not necessarily represent those of their affiliated organizations, or those of the publisher, the editors and the reviewers. Any product that may be evaluated in this article, or claim that may be made by its manufacturer, is not guaranteed or endorsed by the publisher.

## References

[1] Shelton GD Cardinet GH. Bandman E. Expression of fiber type specific proteins during ontogeny of canine temporalis muscle. Muscle Nerve. (1988) 11:124–32. 10.1002/mus.880110207 3343987

[2] Shelton GD Cardinet GH Bandman E. Canine masticatory muscle disorders: a study of 29 cases. Muscle Nerve. (1987) 10:753–66. 10.1002/mus.880100812 3317035

[3] Evans J Levesque D Shelton GD. Canine inflammatory myopathies: a clinicopathologic review of 200 cases. J Vet Intern Med. (2004) 18:679–91. 10.1111/j.1939-1676.2004.tb02606.x 15515585

[4] Castejon-Gonzalez AC Soltero-Rivera M Brown DC Reiter AM. Treatment outcome of 22 dogs with masticatory muscle myositis (1999–2015). J Vet Dent. (2018) 35:281–9. 10.1177/0898756418813536

[5] Melmed C Shelton GD Bergman R Barton C. Masticatory muscle myositis: pathogenesis, diagnosis, and treatment. Compendium Continuing Edu Practis Vet North Am Ed. (2004) 26:590–605.30373608

[6] Orvis J Cardinet III GH. Canine muscle fiber types and susceptibility of masticatory muscles to myositis. Muscle Nerve. (1981) 4:354–9. 10.1002/mus.880040411 7254235

[7] Bubb WJ Sims MH. Fiber type composition of rostral and caudal portions of the digastric muscle in the dog. Am J Vet Res. (1986) 47:1834–42. 2944460

[8] Shelton GD. From dog to man: the broad spectrum of inflammatory myopathies. Neuromuscul Disord. (2007) 17:663–70. 10.1016/j.nmd.2007.06.466 17629703

[9] Gatineau M El-Warrak AO Marretta SM Kamiya D Moreau M. Locked jaw syndrome in dogs and cats: 37 cases (1998–2005). J Vet Dent. (2008) 25:16–22. 10.1177/089875640802500106 18512621

[10] Pitcher GD Hahn CN. Atypical masticatory muscle myositis in three cavalier King Charles spaniel littermates. J Small Anim Pract. (2007) 48:226–8. 10.1111/j.1748-5827.2006.00242.x 17381768

[11] Cauduro A Paolo F Asperio RM Rossini V Dondi M Simonetto LA. Use of MRI for the early diagnosis of masticatory muscle myositis. J Am Anim Hosp Assoc. (2013) 49:347–52. 10.5326/JAAHA-MS-5915 23861264

[12] Paciello O Shelton GD Papparella S. Expression of major histocompatibility complex class I and class II antigens in canine masticatory muscle myositis. Neuromuscul Disord. (2007) 17:313–20. 10.1016/j.nmd.2007.01.012 17360184

[13] Podell M. Inflammatory myopathies. Vet Clin North Am Small Anim Pract. (2002) 32:147–67. 10.1016/S0195-5616(03)00083-4 11785727

[14] De Lahunta A Glass E Kent M. Veterinary Neuroanatomy and Clinical Neurology 4th Edition. St. Louis: Elsevier Editor (2015). Chapter 6, p. 162–196.33634751

[15] Tveterås K Kristensen S. The aetiology and pathogenesis of trismus. Clin Otolaryngol Allied Sci. (1986) 11:383–7. 10.1111/j.1365-2273.1986.tb00141.x 3536195

[16] Cray MT Spector DI West CL. Acute masticatory muscle compartmental syndrome in a dog. J Am Vet Med Assoc. (2018) 253:606–10. 10.2460/javma.253.5.606 30110212

[17] Hermanson JW. The Muscular System. In: Evans HE, De Lahunta A, editors. Miller's Anatomy of the Dog 4th Edition. St. Louis: Elsevier Editor (2013). p. 185–280.

[18] Reiter AM Schwarz T. Computed tomographic appearance of masticatory myositis in dogs: 7 cases (1999–2006). J Am Vet Med Assoc. (2007) 231:924–30. 10.2460/javma.231.6.924 17867978

[19] Bishop TM Glass EN De Lahunta A Shelton GD. Imaging diagnosis—masticatory muscle myositis in a young dog. Vet Radiol Ultrasound. (2008) 49:270–2. 10.1111/j.1740-8261.2008.00364.x 18546784

[20] Platt SR McConnell JF Garosi LS Ladlow J de Stefani A Shelton GD. Magnetic resonance imaging in the diagnosis of canine inflammatory myopathies in three dogs. Vet Radiol Ultrasound. (2006) 47:532–7. 10.1111/j.1740-8261.2006.00181.x 17153060

[21] Schulze M Kötter I Ernemann U Fenchel M Tzaribatchev N Claussen CD. findings in inflammatory muscle diseases and their noninflammatory mimics. AJR Am J Roentgenol. (2009) 192:1708–16. 10.2214/AJR.08.1764 19457839

[22] Nanai B Phillips L Christiansen J Shelton GD. Life threatening complication associated with anesthesia in a dog with masticatory muscle myositis. Vet Surg. (2009) 38:645–9. 10.1111/j.1532-950X.2009.00515.x 19573068

[23] Lei H Yu B Huang Z Yang X Liu Z Mao X. Comparative analysis of mesenchymal stem cells from adult mouse adipose, muscle, and fetal muscle. Mol Biol Rep. (2013) 40:885–92. 10.1007/s11033-012-2129-3 23070912

[24] Sousa-Victor P García-Prat L Serrano AL Perdiguero E Muñoz-Cánoves P. Muscle stem cell aging: regulation and rejuvenation. Trends Endocrinol Metab. (2015) 26:287–96. 10.1016/j.tem.2015.03.006 25869211

