# Supplementary material for: Case report: Atypical and chronic masticatory muscle myositis in a 5-month old Cavalier King Charles Spaniel. Clinical and diagnostic findings, treatment and successful outcome
Source: Front Vet Sci. 2022 Sep 14;9:955758. doi: 10.3389/fvets.2022.955758 (PMC9516294; doi:10.3389/fvets.2022.955758)
Supplement: Supplementary file 3 [file Data_Sheet_1.PDF]

CARE
case report guidelines

CARE Checklist of information to include when writing a case report

(cc) BY-NC-ND

Topic	Item	Checklist item description	Reported on Line
Title	1	The diagnosis or intervention of primary focus followed by the words "case report" . The word case report is reported at the beginning of the title as indicatedin the author guidlines, section article type , case report.	Title section
Key Words	2	2 to 5 key words that identify diagnoses or interventions in this case report, ~~including "case report"~~ . . .	38
Abstract (no references)	3a	Introduction: What is unique about this case and what does it add to the scientific literature?	35-37
	3b	Main symptoms and/or important clinical findings	28-33
	3c	The main diagnoses, therapeutic interventions, and outcomes	33-35
	3d	Conclusion—What is the main "take-away" lesson(s) from this case?	35-37
Introduction	4	One or two paragraphs summarizing why this case is unique (**may include references**)	72-74
Patient Information	5a	De-identified patient specific information.	76-78
	5b	Primary concerns and symptoms of the patient.	76-82
	5c	Medical, family, and psycho-social history including relevant genetic information	Not known
	5d	Relevant past interventions with outcomes	76-78
Clinical Findings	6	Describe significant physical examination (PE) and important clinical findings.	78-82
Timeline	7	Historical and current information from this episode of care organized as a timeline	-
Diagnostic Assessment	8a	Diagnostic testing (such as PE, laboratory testing, imaging, surveys).	91-114
	8b	Diagnostic challenges (such as access to testing, financial, or cultural)	None
	8c	Diagnosis (including other diagnoses considered)	112-114
	8d	Prognosis (such as staging in oncology) where applicable	117-130
Therapeutic Intervention	9a	Types of therapeutic intervention (such as pharmacologic, surgical, preventive, self-care)	114-117; 121-125
	9b	Administration of therapeutic intervention (such as dosage, strength, duration)	114-117; 121-125
	9c	Changes in therapeutic intervention (with rationale)	115-117, 121-125
Follow-up and Outcomes	10a	Clinician and patient-assessed outcomes (if available)	125-130
	10b	Important follow-up diagnostic and other test results	125-130
	10c	Intervention adherence and tolerability (How was this assessed?)	117-121; 125-130
	10d	Adverse and unanticipated events	115-117
Discussion	11a	A scientific discussion of the strengths AND limitations associated with this case report	132-178
	11b	Discussion of the relevant medical literature **with references**.	132-138; 162-171; 175-178
	11c	The scientific rationale for any conclusions (including assessment of possible causes)	134-143; 153-161; 171-172; 179-181
	11d	The primary "take-away" lessons of this case report (without references) in a one paragraph conclusion	181-186
Patient Perspective	12	The patient should share their perspective in one to two paragraphs on the treatment(s) they received	-
Informed Consent	13	Did the patient give informed consent? Please provide if requested	Yes [X] No [ ]
